# Insights into the gelatinization of potato starch by *in situ*^1^H NMR

**DOI:** 10.1039/d1ra08181k

**Published:** 2022-01-26

**Authors:** Yue Wang, Yunxiang Ma, Xudong Gao, Zhipeng Wang, Shenggui Zhang

**Affiliations:** College of Food Science and Engineering, Gansu Agricultural University Lanzhou 730070 Gansu China mayx@gsau.edu.cn zhangshenggui@gsau.edu.cn; Gansu Provincial Key Laboratory of Arid Land Crop Science Lanzhou 730070 China; Chinese Academy of Sciences Key Laboratory of Chemistry of Northwestern Plant Resources, Lanzhou Institute of Chemical Physics, Chinese Academy of Sciences (CAS) Lanzhou 730000 Gansu China; State Key Laboratory of Applied Organic Chemistry, College of Chemistry and Chemical Engineering, Lanzhou University Lanzhou 730000 Gansu China

## Abstract

The gelatinization of potato starch and the effect of NaCl on starch gelatinization were monitored successfully *in situ* by ^1^H NMR spectroscopy. Variable temperature (VT) ^1^H NMR measurement, from 316 K to 340 K, was conducted on a suspension of potato starch and deuterium water as well as a mixture of potato starch, NaCl and deuterium water. The hydration level of starch was determined with the increase of temperature. In the presence of NaCl, the initial gelatinization temperature of potato starch was decreased from 331 to 328 K. Meanwhile, *in situ*^1^H NMR spectroscopy as a function of time was also carried out to monitor the gelatinization with a time resolution of 90 s per spectrum. Furthermore, the effect of using different processing methods during gelatinization, including varying the temperature or time duration, was investigated in detail. It was confirmed that protons from different groups of starch showed different accessibility for water during hydration of starch granules. In comparison with temperature, gelatinization time as the major factor for reaching complete gelatinization was confirmed. We expect that this research, as a continuing effort to apply NMR spectroscopy for characterizing starch, will pave a new way in the structural elucidation of starch.

## Abstract 2

The gelatinization of potato starch and the effect of NaCl on starch gelatinization were monitored successfully *in situ* by ^1^H NMR spectroscopy.

## Graphical abstract

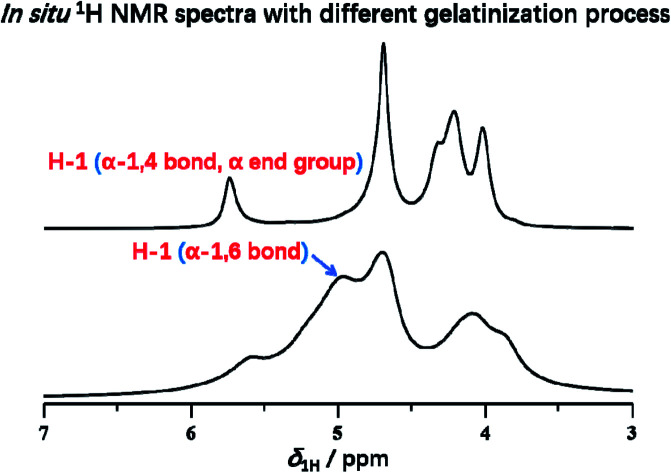

## 1. Introduction

As one of the most abundant and attractive natural biopolymers, starch is the main constituent used to manipulate the quality of food in the human diet, and can be widely found in various botanical sources such as potato, corn, wheat and so on.^1–6^ The structure of starch has been investigated extensively during the last decades. It is commonly considered that native starch is a mixture, which consists of linear amylose and highly branched amylopectin.^7–12^ The difference in amounts and ratio of these two polymers organized within starch granules, gives rise to considerable variability in properties, such as swelling, pasting and gelatinization behaviours.^13–16^ Gelatinization, when starch was heated in water, is one of the most significant processes in the industrial application and development of starch.^17–19^ Under this condition, starch granules undergo disruption of crystallinity and molecular organization. When heating starch in excess water is continued, the granules will suffer an irreversible phase transition, commonly referred to gelatinization.^20–25^ It is generally considered that the functional properties of starch are mainly determined by the extents and effects of gelatinization treatments. Besides, the functionality of starch is intimately related to its hydration levels. A detailed understanding of the hydration of starch will be a significant asset in developing new starch materials with well-designed functionalities.^25,26^ Besides, adding salts is a common process to make the starchy derivatives as normal. It has also been proved that salts such as sodium chloride (NaCl) had a significant effect on the gelatinization of starch.^27^ The effect of sodium chloride could affect the temperature and enthalpy of gelatinization, and simultaneously change the rate and degree of gelatinization.^28,29^ Therefore, it is of importance to understand the detailed gelatinization process where starch is subjected to sodium chloride solution. However, the gelatinization mechanisms in the presence of sodium chloride are still not well understood. In order to manufacture the renewable biomaterials and obtain high quality of foods, it is necessary to get an improved understanding of starch gelatinization and the effect of salts on gelatinization at the molecular level.

Considerable effort has been devoted to monitor the process and discovered the mechanism of gelatinization, using a variety of microscopic, diffraction and spectroscopic techniques, including scanning electron microscope (SEM), powder X-ray diffraction (XRD), differential scanning calorimetry (DSC), Fourier transform infrared (FT-IR) and NMR spectroscopy.^29–37^ However, most of these methods were carried out depending on the post treatment, which usually processed samples with several preparation steps. This will result in the detailed process of gelatinization cannot be monitored carefully in real-time. Generally, it was considered these *ex situ* characterization methods were challenging, due to high sensitivity to sample preparation and limited lifetimes of crystalline structural disruption during gelatinization. Fortunately, the *in situ* measurements, possessing the powerful performance in real-time monitoring could make up this shortage.^18,19,38–43^ For example, rapid visco analysis (RVA), monitoring the process *in situ*, has been widely applied to investigate the gelatinization of starch.^38^ Recently, waxy and high-amylose maize starch gelatinization process was monitored *in situ via* synchrotron XRD in combination with a diamond anvil cell at very high pressures.^41^ Meanwhile, the gelatinization processes of starches from a variety of plant sources were *in situ* observed by a method of polarizing microscope in combination with a hot stage.^42^ All of these techniques studied the process *via* the difference in macroscopic property during starch gelatinization. The detailed structural characterization during the hydration of starch on atomic level remains to be fully studied. However, to the best of our knowledge, there is still few studies employed *in situ* techniques at atomic-levels to monitor the process of starch gelatinization so far. Variable temperature (VT) NMR spectroscopy, which has been widely applied for investigation of structural and phase transition of materials at atomic-levels, undoubtedly is the suitable technique of choice for probing the gelatinization of starch granules.

NMR spectroscopy is a non-invasive and *in situ* method for monitoring the detailed process and structural changes that occur in chemical or non-chemical transition.^44,45^ Compared with other characterization measurements, NMR could acquire the information of structural transition while heating the samples, which makes it possible to probe the transformation process only taken place in high temperature. It was known that starch gelatinization was such a process occurred above gelatinization temperature. Besides, in comparison with the studies of gelatinization using other techniques,^46^ NMR spectroscopy could achieve the detailed information of phase transition process at molecular level.^47^ And on this basis, *in situ* NMR spectroscopy strengthens characterization technique, which makes it possible to real time detect the evolution of hydrogen bonds during gelatinization. Therefore, we envisioned that VT NMR spectroscopy may work as a powerful analytical technique to real time observe the gelatinization process *in situ*.

In this study, we expected to extend the research utilizing *in situ* VT ^1^H NMR spectroscopy to real time monitor the gelatinization process of potato starch. VT ^1^H NMR measurement, from 316 K to 340 K, was carried out on the suspensions of potato starch and deuterium water with the weight ratio of 1 : 7.5. The details of gelatinization were monitored under the treatment of increasing temperature. The hydrogen bonding interaction between the hydroxyl and hydrogen atoms of starch and water was formed gradually with the increase of temperature. Meanwhile, *in situ*^1^H NMR spectra as a function of time (from 0 min to 81 min) were also carried out to monitor the detailed structural transition at atomic level during gelatinization at 328 K. We found that the peak intensity of α-1,6 glucosidic bonds increased obviously with the extending of heating time gradually. Furthermore, the effect with different processing methods during gelatinization, including varying temperature or heating time, was investigated in details. Under different treatment, it was verified amylose and amylopectin showed the different accessibility for water during gelatinization. It was also found that the addition of NaCl decreased the initial temperature of starch gelatinization.

## 2. Materials and methods

### 2.1. Materials

Potato starch was purchased from Shanghai Yuanye Biological Reagent Co., Ltd. Unless otherwise stated, all other reagents were of analytical grade.

### 2.2. ^1^H NMR measurements

Liquid ^1^H NMR spectra were recorded in D_2_O solution at the frequency of 400.06 MHz on a Bruker Avance III 400 MHz NMR spectrometer. Potato starch powder was suspended with D_2_O in a weight ratio of 1 : 7.5. Samples were prepared by weighing *ca.* 80 mg of starch into a NMR tube, then *ca.* 600 mg of D_2_O were added to reach the exact ratio of 1 : 7.5. The starch/sodium chloride solution systems were prepared by weighing *ca.* 80 mg of starch, 25 mg NaCl, and 600 mg of D_2_O into a NMR tube. The variable-temperature NMR measurements were carried out in the range of 316–340 K with the same probe using a Bruker temperature controller. The samples were heated at the target temperature in steps and kept equilibration for approximately 10 minutes before data acquisition. The experiments were recorded with the π/2 pulse of 14 μs, the repeated scans of 16, and the recycle delay of 1 s respectively. Each of the ^1^H NMR measurements takes one and a half minutes without the time for varying temperature. The ^1^H chemical shifts were referenced to the signal of DSS.

## 3. Results and discussion

### 3.1. Variable temperature ^1^H NMR spectra of potato starch

**Fig. 1:**
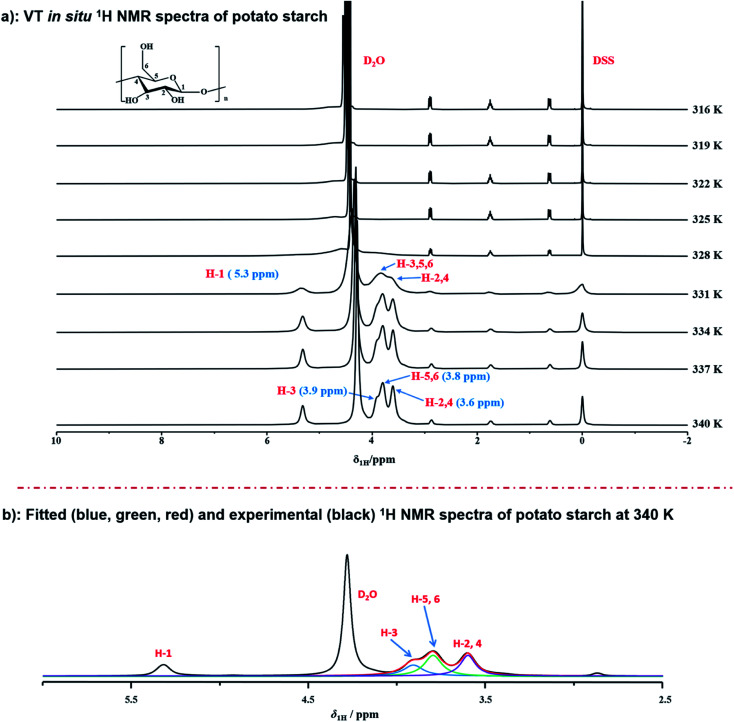
(a) Variable temperature *in situ*^1^H NMR spectra of potato starch at the range of 316–340 K; (b) fitted (blue, purple, and red) and experimental (black) ^1^H NMR spectra of potato starch during gelatinization at 340 K.

To real-time monitor the detailed gelatinization process of potato starch *in situ* at atomic-levels, the variable temperature ^1^H NMR measurements at *T* = 316–340 K were conducted upon the suspensions of potato starch and deuterium water with the weight ratio of 1 : 7.5 (Fig. 1a). Under this condition of data acquisition, the proton NMR signal of starch, only dissolved or partially hydrated into water, can be observed in ^1^H NMR spectra. Instead, protons undissolved or inaccessible to water are not detected, due to the strong ^1^H homonuclear dipole–dipole interactions. The assignment of ^1^H NMR chemical shifts from starch was referred to the previous papers.^48–51^ It should be noted that no hydroxyl protons were detected during the dissolved starch in D_2_O, due to the effective exchange between OH and OD.^51^ It was found that only the peak of deuterium water (at 4.5–4.7 ppm) could be observed obviously, which indicated that the gelatinization process did not occur at 316 K. When the temperature was increased to 331 K, a broad signal (centred at 5.3 ppm) was recorded at down-field, due to the ^1^H homonuclear dipole–dipole interactions. We speculated that this peak may be derived from H-1, which were attributed to both the protons of α-1,4 bond and α-end group in starch, and this result was consistent with the Larsen's work.^51^ They applied the ^1^H high-resolution MAS NMR spectroscopy to characterize the signals from α-1,4 bond (5.4 ppm) and α-end group (5.2 ppm) of potato starch, respectively. This suggested that the end groups including α-1,4 glucosidic backbone and α-end group were easily accessible for water instead of other groups in starch. It should be noted that only the protons located in mobile regions are detected *via*^1^H NMR. Instead, protons from immobile starch regions do not contribute to ^1^H NMR spectrum. Hence, the strong peak centred at 5.3 ppm indicated that α-1,4 bond and α-end group were located on the mobile regions. The absence of protons from α-1,6 backbone was detected, which was consistent pioneer's work.^52^ It has been proved that protons α-1,6 backbone was stabilized by the presence of structural water at a low degree of gelatinization, which strongly increased the dipole–dipole interactions and do not contribute to the ^1^H NMR spectra. Meanwhile, a new broad signal from the H-2,3,4,5,6 of starch was observed, indicating that the interaction between protons of H-2,3,4,5,6 in starch and water molecules began to occur at 331 K. As the temperature increased, for example at 340 K, the peak width at half height of H-1 was decreased from 90 Hz to 34 Hz, which was attributed to the higher extent of hydration. Besides, it was considered that amylose has the more reducing end groups than amylopectin, due to the lower molecular mass of amylose. Hence, the signal of H-1 also demonstrated that the end groups of amylose showed the better accessibility and higher degree of hydration for water than amylopectin. Furthermore, as the temperature increased to 334 K, two well-resolved peaks of H-2,3,4,5,6 centered at 3.6 and 3.8 ppm were observed, which were from the signals of H-2,4 and H-3,5,6 respectively. This observation suggested that the gelatinization process of potato starch was started at 331 K. When the temperature increased to 337 K, the peak of H-3,5,6 split into two sub-peaks caused by further progress of the gelatinization, and these two sub-peaks become well resolved as temperature increased to 340 K with chemical shifts of 3.8 and 3.9 ppm, which were attributed to the group of H-5,6 and H-3, respectively. It was also indicated that this suspension was hydrated to the large extent of gelatinization. In order to better distinguish these two peaks of H-5,6 and H-3, as displayed in Fig. 1b, two peaks were fitted as the green (peak of H-5,6) and blue line (peak of H-3) by DMfit software,^53^ respectively. Meanwhile, the ^1^H NMR spectrum at 340 K was almost consistent with that of 337 K, indicating that the suspension of potato starch and deuterium water with the weight ratio of 1 : 7.5 might be gelatinized to reach upper limit to the extent of gelatinization.

**Fig. 2:**
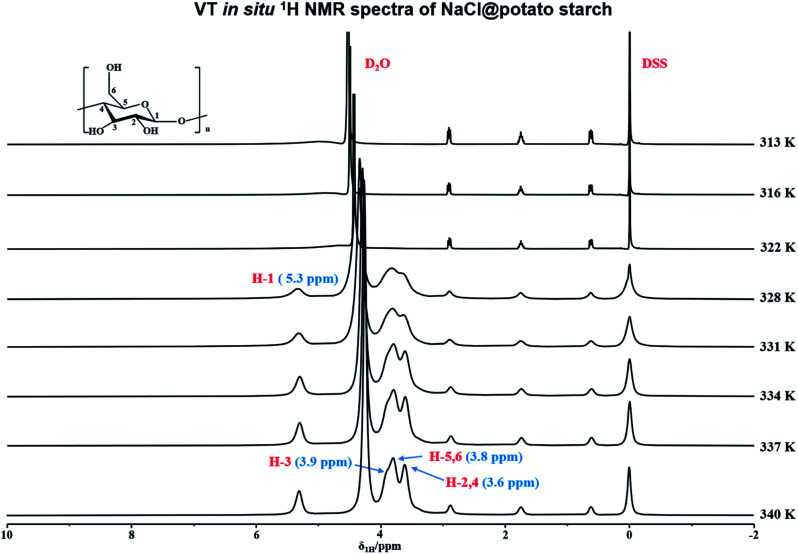
Variable temperature *in situ*^1^H NMR spectra of NaCl@potato starch at the range of 316–340 K.

In order to *in situ* monitor the gelatinization of potato starch in the presence of NaCl, the variable temperature ^1^H NMR at *T* = 316–340 K were carried out upon the sample of potato starch, NaCl and deuterium water (donated as NaCl@potato starch). It was found that the ^1^H NMR spectra of NaCl@potato starch were consistent with that of potato starch during the whole variable temperature process. As shown in Fig. 2, the initial gelatinization temperature of potato starch in the presence of NaCl was 328 K, but the initial gelatinization temperature of potato starch without NaCl was 331 K detected by variable temperature ^1^H NMR (seen in Fig. 1). This result suggested the damage of starch granules was accelerated, which gave rise to the well hydration of starch with D_2_O.

### 3.2. *In situ* ^1^H NMR spectra of potato starch as a function of time during gelatinization

**Fig. 3:**
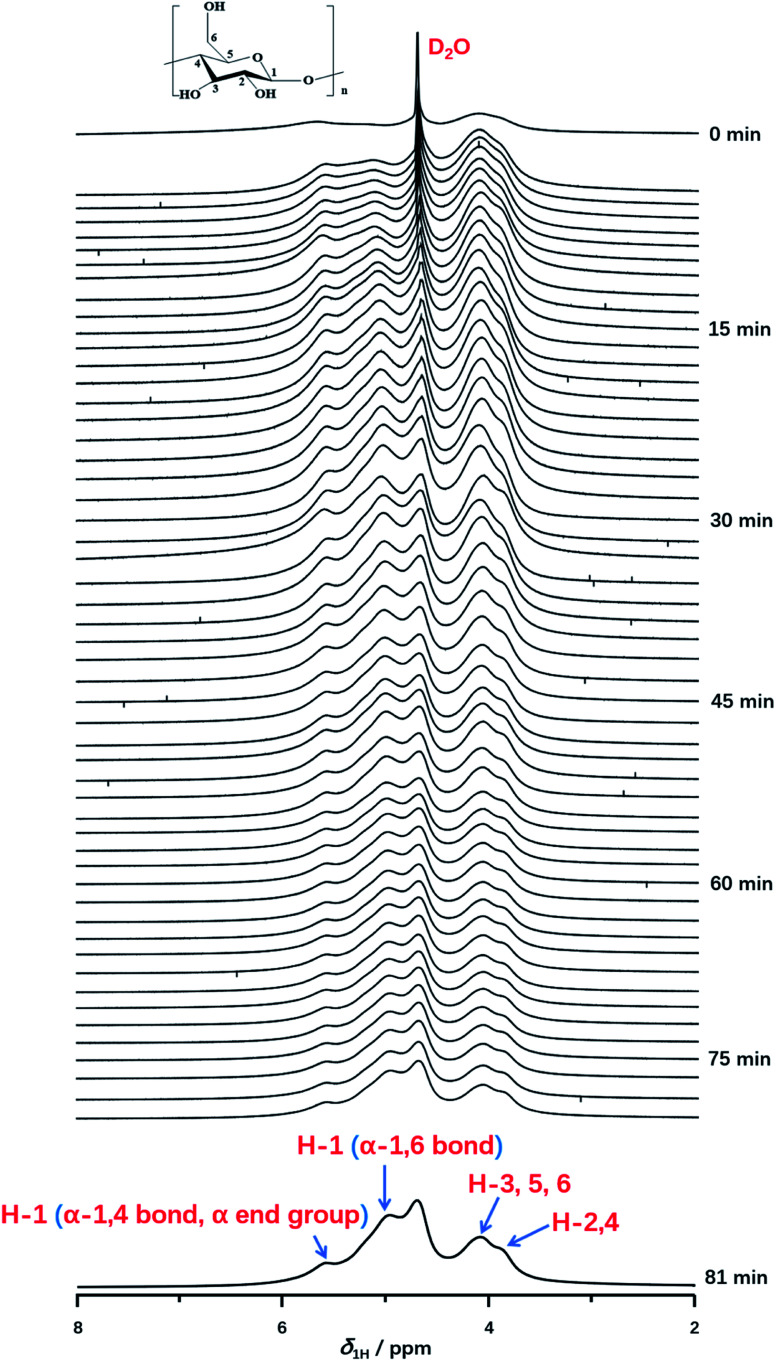
*In situ* ^1^H NMR spectra as a function of time recorded with the time resolution of 90 s per spectrum during gelatinization of potato starch at 331 K from 0 to 81 min.

Furthermore, *in situ*^1^H NMR spectra as a function of time (from 0 min to 81 min) were carried out to monitor the detailed structural transition at atomic level during gelatinization as shown in Fig. 3. Each experiment was recorded with the time resolution of 90 s per each spectrum at 331 K. At the time of 0 min, the peak of deuterium water with considerable intensity was observed obviously. Instead, ^1^H NMR signals of starch cannot be resolved unambiguously, which indicated the starch hydration has not occurred yet. After the first measurement, ^1^H NMR signals of starch protons could be detected obviously, which was attributed a small amount of starch begins to dissolve in deuterium water and the gelatinization occurred. As the increase of the experimental time, the signal intensity of starch was increased, due to the well solubility of starch at high temperature. This indicated starch gelatinization happened within a large extent. At experimental time of 45 min, the well resolved ^1^H NMR can be recorded in comparison with that of 0 min. Referred to the previous works,^49,51^ peaks at 3.8 and 4.0 ppm were attributed to H-2,4 and H-3,5,6 of starch. As shown in Fig. 3, the distorted peaks centered at 5.0 and 5.6 ppm were attributed to H-1 peak from α-1,6 glucosidic bonds, α-end group and α-1,4 glucosidic backbone, respectively. Interestingly, the H-1 signal of α-1,6 glucosidic bonds was observed, which suggested the stabilization of α-1,6 bonds, caused by structural water, was destroyed at high enough degree of gelatinization. Thus, α-1,6 bonds can be accessible for water along with the extending heating time. This result also indicated a high degree of gelatinization had already been reached. Besides, we found that the intensity of α-1,6 bonds was much higher than that of α-end group and α-1,4 bond. At an enough extent of gelatinization, starch granule regions, which was previous inaccessible for water began to dissolve with the increase of heating time. And the content of amylopectin is much higher than amylose in potato starch. Large amount of amylopectin can be dissolved in D_2_O than that of amylose. Hence, the stronger intensity of α-1,6 bonds was observed than that of α-end group and α-1,4 bond. No other ^1^H NMR peak occurred until the end of the experiments (seen in Fig. 3, spectrum of 81 min). Furthermore, we found that the spectra with the heating time from 75 to 82.5 min were consistent with that of 75 min, which indicated the starch was completely gelatinized after 75 minutes.

**Fig. 4:**
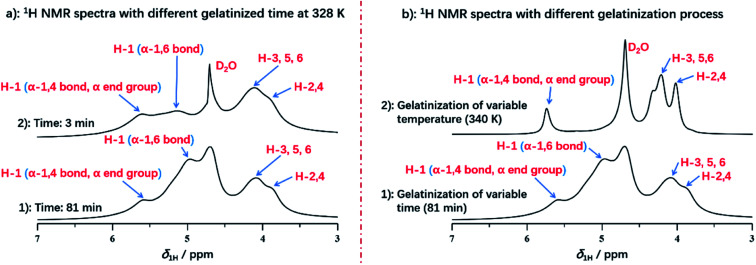
(a) ^1^H NMR spectra of starch with different gelatinized time at 331 K; (b) ^1^H NMR spectra of starch with different treatment during gelatinization.

Interestingly, we compared the ^1^H NMR spectra of variable heating time at 3 min and 82.5 min as displayed in Fig. 4a. The H-1 peak (α-1,4 bond and α end group) and H-2,3,4,5,6 from spectrum at 3 min was almost consistent with that of the spectrum at 82.5 min, which indicated that protons from H-2,3,4,5,6, α-1,4 bond and α end group were located in the mobile regions, and were accessible to deuterium water easily at the beginning of gelatinization. However, it was noted that the intensity of α-1,6 glucosidic bonds was increased obviously with the gelatinization time in comparison with that of α-1,4 bonds. At heating time of 3 min, H-1 peak of α-1,6 glucosidic bonds with low intensity can be detected, with the increase of heating time (for example at 81 min seen in Fig. 4a), the peak intensity increased obviously, which was resulted from the stabilization of α-1,6 bonds by structural water.^52^ At the beginning, α-1,6 bonds were stabilized by structural water due to the low degree of gelatinization. This resulted in the strong dipolar interactions, which caused no contribution to the spectrum. However, with increasing heating time, this type of stabilized effect was broken at the high enough extent of gelatinization, which diminished the dipolar interaction. Therefore, the peak of α-1,6 bonds could be detected and intensity was increased with gelatinization time.

It was also found that different processing methods, including varying temperature or extending heating time, showed the significant impact on the details of starch gelatinization. As displayed in Fig. 4b, we compared two spectra with different processing methods, both of which were selected at the end of each gelatinization process. The spectrum of variable temperature at 340 K has the smaller full width at half maximum in each peak than that of heating time at 81 min, due to the better solubility. This demonstrated that the well solubility can be obtained by the treatment of increasing temperature instead of extending gelatinization time. And temperature has more significant effect rather than the heating time during gelatinization of potato starch. However, compared with treatment of extending time, the absence of H-1 peak from α-1,6 glucosidic bonds was detected in the spectrum of gelatinization under increasing temperature, which indicated that treatment of increasing temperature did not benefit to reach the higher extent of gelatinization. Instead, gelatinization time may be the major factor for acquisition of completely gelatinization. It was also verified that amylopectin has the poor ability of hydration than amylose, due to its water-inaccessible regions.

**Fig. 5:**
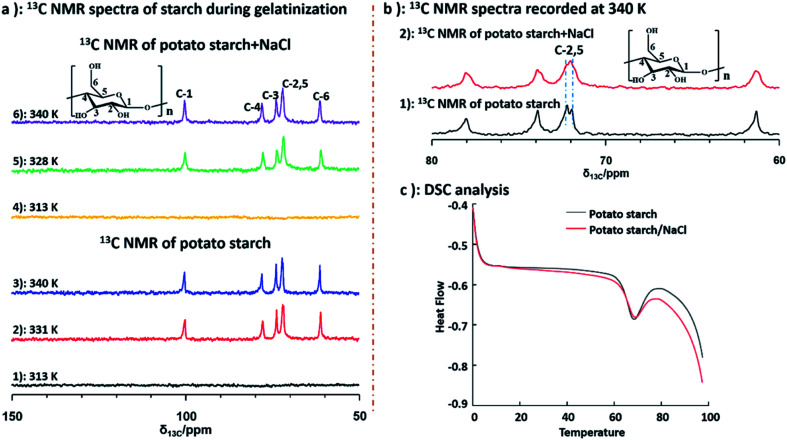
(a) ^13^C NMR spectra of starch and starch/NaCl at different temperature; (b) ^13^C NMR spectra of starch recorded at 340 K; (c) DSC data of starch and starch/NaCl.

Furthermore, to better understand the structural changes of starch backbone, ^13^C NMR spectra at 313 K, 331 K and 340 K for potato and deuterium sample, as well as the sample of potato and NaCl water at 313 K, 328 K and 340 K were measured by liquid state NMR respectively. Due to poor solubility of starch, no NMR signal was recorded in the spectra of both potato starch and potato starch/NaCl sample at 313 K. Importantly, when the temperature was increased to 328 and 331 K, samples of starch and starch/NaCl began to dissolve in D_2_O respectively. As a result, the peaks of starch could be detected as shown in Fig. 5 (a2 and a5). The well resolved ^13^C NMR spectra can be obtained when the temperature increased to 340 K. The ^13^C NMR chemical shifts for starch were assigned according to the literature.^54^ Chemical shifts of starch was consistent with that of starch/NaCl during gelatinization, this indicated that no structural changes of starchy backbone occurred with and without adding NaCl. Furthermore, from Fig. 5b, the well resolved C-2,5 peaks of starch were observed obviously, instead only one broad peak of C-2,5 was detected in sample of starch/NaCl at 340 K. This broad signal was attributed to the amorphous structure of starch, which caused by the higher degree of gelatinization. However, two well resolved peaks of C-2,5 indicated the maintain of crystalline structure due to its low degree of gelatinization. This result demonstrated that the effect of NaCl could give rise to the higher degree of gelatinization. Differential scanning calorimetry (DSC) measurement was also used to characterize the gelatinization of potato starch and potato starch/NaCl. As shown in Fig. 5c, the peak temperature, determined as 67.98 and 67.28 °C for samples of starch and starch/NaCl, were in agreement with literature.^55^ This result was almost consistent with that of ^1^H NMR spectroscopy. It indicated that a large amount of starch begins to melt and dissolve in water. As a result, a well resolved ^1^H and ^13^C NMR spectra could be recorded under this condition.

## 4. Conclusion

In conclusion, we have successfully applied ^1^H NMR spectroscopy to *in situ* monitor the gelatinization process of potato starch at molecular level. VT ^1^H NMR measurement, from 316 K to 340 K, was measured on the suspension of potato starch and deuterium water with the weight ratio of 1 : 7.5. The hydration level of starch was determined with the increase of temperature. To probe the effect of NaCl on starch gelatinization, VT ^1^H NMR measurement was carried out on the suspension of potato starch, NaCl and deuterium water. In the presence of NaCl, the initial gelatinization temperature of potato starch was decreased from 331 to 328 K. Meanwhile, *in situ*^1^H NMR spectra as a function of time were also carried out to monitor the detailed process during gelatinization. We found that the peak intensity of α-1,6 glucosidic bonds increased obviously with the extending of heating time gradually. Furthermore, the effect with different processing methods during gelatinization, including varying temperature or extending heating time, was investigated in details. Under different treatment, it was verified protons from different group of starch showed the different accessibility for water during gelatinization. In comparison with temperature, gelatinization time as the major factor for acquisition of completely gelatinization was confirmed. All these results indicated that ^1^H NMR spectroscopy indeed works as a premier tool for *in situ* monitoring the gelatinization of starch. We expect that this research, as a continuing effort to apply NMR spectroscopy^16,56,57^ for characterizing starch will pave a new way in the structural elucidation of starch.

## Conflicts of interest

The authors declare no competing financial interest.

## Supplementary Material
